# Editorial: The role of normal blood lipid levels in metabolic and endocrine diseases

**DOI:** 10.3389/fendo.2026.1910041

**Published:** 2026-07-03

**Authors:** Rubing Guo, Wei Zhao

**Affiliations:** 1 Department of Clinical Laboratory, China-Japan Friendship Hospital, Beijing, China; 2 Department of Clinical Laboratory, Quanzhou Orthopedic-Traumatology Hospital, Quanzhou, China

**Keywords:** dose-response relationship, endocrine diseases, insulin resistance, metabolic diseases, normal blood lipid levels

## Introduction

Metabolic and endocrine diseases—including type 2 diabetes, prediabetes, hyperuricemia, and obesity-related disorders—continue to impose a growing global health burden (1–3). Despite advances in prevention and management, these conditions remain major contributors to cardiovascular morbidity and mortality (4). Blood lipids play a fundamental dual role in human physiology: they are essential for cellular membrane integrity, hormone synthesis, energy homeostasis, and endocrine signaling (5). However, even modest fluctuations within clinically defined “normal” reference ranges can influence metabolic risk in ways that traditional binary thresholds fail to capture (6).

Conventionally, clinical lipid management has relied on rigid threshold-based approaches that classify individuals as “normal” or “abnormal” (7). This dichotomous approach, while useful for population-level screening, may obscure a more nuanced reality: strictly controlling lipid fluctuations within the normal reference range can also modulate disease risk (8). Mounting evidence indicates that the relationship between lipids and metabolic health is not a simple all-or-none threshold phenomenon. Instead, even subtle variations in certain lipid components among individuals with “normal” levels can exhibit dose–response associations with outcomes such as type 2 diabetes and insulin resistance (8, 9). Static normal values may therefore mask individual risk gradients, calling for a paradigm shift from target-based management toward refined, continuous risk assessment.

This Research Topic assembles rigorous investigations that illuminate the multifaceted relationships between normal blood lipid levels and the development or progression of metabolic and endocrine diseases across diverse populations and clinical contexts.

## Early insulin resistance and diabetes risk hidden within normal lipid levels

Multiple contributions demonstrate that even when conventional lipid parameters remain within the normal range, certain indices can effectively unmask residual diabetes risk. Wang et al. and Chen et al. independently show that the triglyceride-glucose (TyG) index—a surrogate marker of insulin resistance—strongly predicts incident diabetes in individuals with normal fasting triglyceride and glucose levels. Notably, both studies identify critical thresholds (TyG > 8.41 and > 8.53, respectively), beyond which diabetes risk escalates exponentially. These findings align with broader evidence from large prospective cohorts confirming the TyG index as a robust predictor of type 2 diabetes even among individuals with normal baseline glucose, with similar inflection points around 8.3–8.7 in various populations (10).

Extending this logic to low-density lipoprotein cholesterol (LDL-C), Tian et al. report that even within the normal range (<3.4 mmol/L), each incremental increase in LDL-C is associated with higher diabetes risk, with a potential safety threshold around 1.8 mmol/L. Perhaps most strikingly, Wang et al. reveal that individuals who have “recovered” from dyslipidemia still carry a 37% higher type 2 diabetes risk compared to those with persistently normal lipids, underscoring that normalization of traditional markers does not equate to metabolic restoration. Together, these findings implicate subclinical insulin resistance and early β-cell lipotoxicity as common mechanistic threads. This residual risk is consistent with observations that prior dyslipidemia leaves lasting metabolic vulnerability, even after lipid normalization (11).

## Remnant cholesterol – a rising player in normal-range risk stratification

Remnant cholesterol (RC), often overlooked in routine assessments, emerges as a surprisingly potent predictor. Liu et al. demonstrate a negative, nonlinear relationship between RC levels and reversion to normoglycemia in non-obese Chinese adults with prediabetes; when RC exceeds 1.10 mmol/L, the likelihood of returning to normal glucose declines sharply. This pattern is consistent with other cohort evidence showing nonlinear inverse associations between RC and glycemic recovery in prediabetes (12).

In a separate cross-sectional study, Xiong and Ma show that RC is dose-dependently associated with hyperuricemia in a normolipidemic population, with no evidence of a threshold—suggesting a linear risk gradient across the entire RC spectrum. Importantly, mediation analysis indicates that insulin resistance accounts for nearly 40% of this association, further linking RC to the insulin resistance–inflammation–urate axis. Similar positive, often linear or dose-dependent associations between RC and hyperuricemia have been reported in general and normolipidemic populations, independent of traditional lipids (13).

## Composite lipid indices and multi-system crosstalk

Beyond single lipid components, composite indices that integrate multiple parameters often outperform traditional markers. Liu et al. report that the lipoprotein combined index (LCI) is positively and linearly associated with hyperuricemia prevalence among normolipidemic oilfield workers, independent of conventional lipid levels. This superior performance of LCI over traditional lipids is consistent with findings in other populations, where composite indices such as LCI show stronger associations with metabolic disturbances like non-alcoholic fatty liver disease and cardiometabolic risk.

Expanding the metabolic network, Tan et al. provide proof-of-concept that an 8-week intervention with Chenpi Jiaosu (a fermented tangerine peel product) significantly lowers triglycerides and body weight while remodeling gut microbiota and serum metabolome—illustrating how natural compounds may modulate normal-range lipid metabolism via the gut-liver axis.

Furthermore, Zhang et al. introduce novel hypoxic metrics (pRED_3p, SBII) from sleep apnea patients, revealing independent, linear associations with multiple glucose and lipid parameters, including composite indices like LCI and visceral adiposity index. This suggests that intermittent hypoxia, even in the absence of overt dyslipidemia, can drive metabolic dysregulation. These links are supported by studies showing that obstructive sleep apnea-related hypoxia independently correlates with disturbances in both glucose/lipid metabolism and composite atherogenic indices (14).

## Trace elements and lipoprotein interactions

Finally, Wu et al. add an intriguing layer by showing that the association between whole-blood copper concentration and glycemic control in type 2 diabetes is significantly modified by apolipoprotein B levels. In individuals with lower apolipoprotein B (apoB), higher copper correlates with worse HbA1c; this relationship disappears in those with higher apoB. This finding hints that lipoproteins may buffer metal-induced oxidative stress, opening new questions about how normal-range lipid profiles modulate trace element toxicity. This interaction aligns with evidence that apolipoprotein B-containing lipoproteins participate in copper-catalyzed LDL oxidation, where higher apoB burden may alter the oxidative environment and mitigate copper’s adverse effects on β-cell function and glycemic control (15).

## Conclusion

Collectively, the contributions to this Research Topic advance our understanding of the nuanced role of normal blood lipid levels in metabolic and endocrine diseases. They demonstrate that even within clinically defined normal ranges, lipid components and composite indices show continuous, dose-dependent, and often nonlinear associations with risks of type 2 diabetes, prediabetes reversion, hyperuricemia, and related metabolic disturbances. Most studies report observational associations rather than direct causality, with underlying mechanisms—including lipotoxicity, insulin resistance, gut microbiota changes, and endocrine dysregulation—requiring further interventional and multi-omics investigation.
